# Consumption of Pornographic Materials among Hong Kong Early Adolescents: A Replication

**DOI:** 10.1100/2012/406063

**Published:** 2012-06-18

**Authors:** Daniel T. L. Shek, Cecilia M. S. Ma

**Affiliations:** ^1^Department of Applied Social Sciences, Faculty of Health and Social Science, The Hong Kong Polytechnic University, Room HJ407, Core H, Hong Kong; ^2^Public Policy Research Institute, The Hong Kong Polytechnic University, Hong Kong; ^3^Department of Social Work, East China Normal University, Shanghai 200062, China; ^4^Kiang Wu Nursing College of Macau, Macau, China; ^5^Division of Adolescent Medicine, Department of Pediatrics, College of Medicine, Kentucky Children's Hospital, University of Kentucky, KY 40506, USA

## Abstract

Consumption of pornographic materials was examined in 3,638 secondary 2 students in Hong Kong. Results showed that over 80% of the respondents had never consumed pornographic materials in the past year. Internet pornography was the most common medium that adolescents used when viewing pornographic materials. Males reported a higher level of pornography consumption than did females. Participants who were born in mainland China were more likely to consume pornographic materials than their Hong Kong counterparts. Regardless of the types of pornographic materials, the levels of pornography consumption significantly increased over time. Results also showed that higher levels of positive youth development and better family functioning were concurrently related to a lower level of pornography consumption at secondary 2. The relative contribution of positive youth development and family factors to pornographic material consumption was also explored.

## 1. Introduction

Research has shown that mass media, particularly the Internet, is one of the most popular channels through which young people obtain sexual information [1–4]. This might be related to the easy accessibility, affordability, and anonymity of the internet [5]. Young people often use the Internet for nonrecreational (e.g., doing homework and project) and recreational purposes (e.g., playing online video games, watching videos, and visiting social networking sites). As prior research on pornography investigating sexually explicit materials mainly focused on the traditional form of print pornography, health practitioners have raised the concern about the potential harms of internet pornography. This is further supported by the growing number of online pornography among youths in the recent studies [6]. Given the fact that early exposure to pornography is a significant risk factor associated with problematic sexual behavior [7–9], early detection of the signs of consumption of sexual materials and exploring the determinants of this behavior is necessary.

Gender has been commonly examined in mainstream pornography research [10–12]. Compared to females, males reported higher levels of pornography consumption, more attracted to hardcore pornography, and more sexually excited after viewing pornography [11, 13]. However, few studies have examined such differences in Chinese samples. Given the majority of studies were conducted in Western contexts, more attention to study the correlates of pornography consumption in non-Western contexts is warranted. There are two reasons why Chinese adolescents should be studied. First, Chinese people constitute roughly one-fifth of the world's population. Second, sex was a “taboo” and socially inhibited in the traditional Chinese culture. With rapid Westernization and modernization, it is expected that there might be some changes in sexual attitudes amongst Chinese adolescents. Hence, there is a need to understand consumption of pornographic materials in adolescents in this area.

Family processes play an important role in adolescent problem behavior. There is evidence that weak parental monitoring and lack of parental warmth and poor family functioning were conducive to adolescent problem behavior [7, 8]. In the local context, Shek [14] also showed that poor parenting and family dysfunction predicted adolescent problem behavior. Similar observations are also shown in young people sexual behavior. Family factors, such as family structure, parental monitoring, and parental trust were associated with young people sexual behavior [15–17]. However, it is not clear whether perceived family functioning is related to pornographic materials consumption. Besides, it would be important to look at how attributes of adolescents such as self-concept, resilience would be related to pornographic material consumption. The present study attempted to address these research gaps.

Given the majority of the pornography studies are crosssectional in nature [13, 18, 19], it would be helpful to study the changes in the pattern of pornography consumption over time. Researchers highlighted the need to understand the interplay of factors related to pornography consumption [20]. As argued by Fisher and Barak [21], future research should focus on “(a) personality characteristics that incline individuals to seek out sexually explicit materials…  (b) the effects of contact with sexually explicit media on individuals who chose to consume such material” (p. 315). In view of the proliferation of sexual information, more attention in this area is needed. This information will provide insights and guidelines regarding the design of effective youth programs that could help young people achieve a sexually healthy adulthood.

Shek and Ma [22] examined the consumption of pornographic materials in 3,328 secondary 1 students in Hong Kong. Results showed that most secondary 1 students had not consumed pornographic materials in the past year. In addition, gender was related to the level of exposure to pornographic materials. Different measures of positive youth development and family functioning were significant predictors to adolescents' consumption of pornographic materials. In the present study, we extended previous research in several ways. First, we examined the profiles of pornography consumption as well as the psychosocial correlates among Chinese adolescents in their secondary 2 school year. Second, to replicate the findings observed at Time 1, we examined the linkages between personal and family factors and consumption of pornographic materials in secondary 2 students. Lastly, we explored the changes of pornography consumption over a year.

## 2. Methodogy

The data reported in this paper were derived from the second wave of a six-year longitudinal study of adolescent development and their families in Hong Kong (Wave 1: academic year 2009-10; Wave 2: academic year 2010-11). The longitudinal study is a component of the extension phase of the project P.A.T.H.S. which is financially supported by the Hong Kong Jockey Club Charities Trust. A total of 3,638 Secondary 2 students (Grade 8) from 28 schools participated in this study. Among the participants, 1,864 (52%) were boys and 1,716 (48%) were girls, with 58 of them did not respond to the question. The mean age of the participants was 13.6 years old (SD = .75). The demographic information of the participants is shown in Table 1.

During data collection, the purpose of the study was mentioned and confidentiality of the collected data was assured. School, parental, and student consent had been obtained prior to data collection. All participants responded to all scales in the questionnaire in a self-administration format. Adequate time was provided for the participants to complete the questionnaire. A trained research assistant was present throughout the administration process.

### 2.1. Instruments

#### 2.1.1. The Chinese Positive Youth Development Scale (CPYDS)

The Chinese Positive Youth Development Scale (CPYDS) [23] was developed to assess positive youth development. The CPYDS has 15 subscales, including bonding (BO), resilience (RE), social competence (SC), recognition for positive behavior (PB), emotional competence (EC), cognitive competence (CC), behavioral competence (BC), moral competence (MC), self-determination (SD), self-efficacy (SE), clear and positive identity (SI), beliefs in the future (BF), prosocial involvement (PI), prosocial norms (PN), and spirituality (SP). The details of the items can be seen in Shek et al. [23]. A 6-point Likert scale (1 = strongly disagree to 6 = strongly agree) was used to assess the responses of the participants.

Using multigroup confirmatory factor analyses (MCFA), Shek and Ma [24] showed that the 15 basic dimensions of the CPYDS could be subsumed under four higher order factors, including cognitive-behavioral competencies (CBC), prosocial attributes (PA), positive identity (PID), and general positive youth development qualities (GPYDQ). Evidence of factorial invariance in terms of configuration, first-order factor loadings, second-order factor loadings, intercepts of measured variable, and intercepts of first-order latent factor, was found. In short, existing research findings showed that the CPYDS is a valid and reliable instrument.

#### 2.1.2. The Chinese Family Assessment Instrument (CFAI)

The Chinese Family Assessment Instrument (CFAI) was used to assess family functioning. In the present study, three subscales, including mutuality (mutual support, love, and concern among family members), communication (frequency and nature of interaction among family members), conflicts and harmony (presence of conflicts and harmonious behavior in the family) were examined. The five response options were “very similar,” “somewhat similar,” “neither similar nor dissimilar,” “somewhat dissimilar,” and “very dissimilar.” A higher total score on the subscales indicated a higher level of positive family functioning. The reliability and validity of the CFAI were supported in previous studies [14, 25–27]. Furthermore, multigroup confirmatory factor analyses (MCFA) showed the existence of two higher order factors (i.e., family interaction and parenting) and factorial invariance of the CFAI across gender and subgroups [28].

#### 2.1.3. Exposure to Pornographic Materials

Twelve items were used to assess the consumption of two types of pornographic materials during the last year. They were internet pornography (e.g., pornographic stories, pictures, videos and websites) and traditional pornography (e.g., pornographic movies, rental films, movies on cable TV, magazines, books and comics). Participants answered on a 6-point Likert scale (0 = never; 1 = less than 1 time a week; 2 = 1–3 times a week; 3 = about 1 time a week; 4 = several times a week; 5 = daily). A composite score was calculated by averaging all twelve-item scores in order to obtain the mean of the overall exposure to pornographic materials. The same method was used to calculate the mean consumption scores of traditional and internet pornographic materials.

#### 2.1.4. Family Background Characteristics

An item was asked to assess whether participants received financial aids (known as CSSA-comprehensive social security assistance) from the government of Hong Kong. For example *“Your family is now receiving CSSA?”* (1 = Yes, 0 = No). Another item was used to assess the immigrant status of the participants (0 = Hong Kong, 1 = mainland China, 2 = others).

### 2.2. Results

Reliability analyses showed that all subscales had acceptable internal consistency at Time 2 (Table 4). The prevalence of pornography consumption among Hong Kong adolescents is shown in Tables 2 and 3. Over 80% of adolescents reported they had never consumed pornographic materials over the past year. The degree of adolescents' exposure to the Internet pornographic materials was significantly higher than those found in the traditional pornographic materials (mean rank = 236.50, *Z *= −18.69, *P* < .01).

A Chi-square (Pearson's test of independence) analysis was performed to examine the relationships between respondents' characteristics (i.e., gender, immigrant status, and family economic background) and pornography consumption. Significant relationships were found for gender and immigrant status but not for family economic background (Table 5). Males were more likely to consume different types of pornographic materials than did females (*χ* ^2^(1, *N* = 3, 580) = 85.68, *P* < .01). Participants who were born in mainland China were more likely to expose to pornography consumption than those who were born in Hong Kong (*χ* ^2^(1, *N* = 3, 601) = 14.92, *P* < .01). It is noteworthy that the relation between family economic background and pornography consumption was not significant.

Analyses based on Pearson correlation showed that all positive youth development and family functioning measures were negatively correlated (ranging from −.05 to −.18) with the overall pornography exposure at Time 2. In general, higher levels of positive youth development and family functioning were related to lower levels of pornography consumption (Table 4).

To further examine whether there was a significant change in the consumption of pornographic materials across time, a series of paired *t*-test was performed (Table 6). Regardless of the types of pornographic materials, higher levels of pornography exposure were found over time (Overall: *t*(2,851) = −7.80, *P* < .01; Internet: *t*(2,830) = −8.59, *P* < .01; Traditional: *t*(2,856) = −3.96, *P* < .01).

## 3. Discussion

The goal of the current study was to examine the prevalence and psychosocial correlates of pornography consumption among secondary 2 students in Hong Kong. Similar to the data collected at the secondary 1 year, most of the participants (over 80%) reported that they had never read or watched pornographic materials in the past 12 months. Compared to the findings in the Western studies [13, 29, 30], the results of our present study were generally lower. There are two possible explanations for this observation. First, it may be related to the fact that this sample was generally younger than those employed in the previous studies. Second, as there is a strong cultural sanction against sexual permissiveness in the Chinese culture, socialization against consumption of pornographic materials might also account for the low levels of exposure to pornography among the Chinese youths.

Consistent with the Wave 1 data, pornographic materials were mostly consumed via the Internet. In particular, there has been a steady increase in the consumption of pornographic materials over a year. During this period of risk-taking, young people are inclined to experiment and engage in problem behaviors, such as substance use and teenage pregnancy in order to find their own identity [31]. The prevalence of pornography is also in line with Ybarra and Mitchell's findings [32] that adolescents reported a higher degree of online pornography exposure than traditional print medium (e.g., magazine, books, and comics). Among them, males (24%) were significantly more likely to consume online pornography than their female counterparts (12%), and this is consistent with prior studies [13, 33]. This result deserves our attention as research findings suggested that young people who viewed online pornography were susceptible to emotional challenge and interpersonal problems [32, 34]. Perhaps, this might be related to the decrease in social interaction with frequent use of the internet. This is further supported by Lam and Chan [35] who examined the potential impact of internet pornography exposure among Chinese young adults. Focusing exclusively on men, they found that prolonged exposure to internet pornography was related to premarital sexual permissiveness and proclivities towards sexual harassment. Given the prevalence of pornography exposure, comprehensive sex education that focuses on the development of correct attitudes and values towards sexual behavior and contemporary pornography is important.

An interesting finding was that immigrant status, but not family socioeconomic background, was associated with pornography consumption. This finding suggests that adolescents' demographic background, rather than their family financial status, plays an important role on their pornography consumption. Furthermore, results showed that mainland Chinese reported higher levels of pornographic exposure than did their Hong Kong counterparts. Perhaps, compared to Hong Kong adolescents, mainland adolescents expressed higher curiosity towards sexuality and stronger needs to satisfy their sexual desire due to the limited availability of pornographic materials, especially via the Internet, in mainland China. Furthermore, it is possible that young people from the mainland may use pornographic consumption as a way to cope with stress. Our findings highlight the need for research specifically examining the sexual background factors and its effects on pornography consumption and individuals' psychological wellbeing.

The present findings support earlier findings of the beneficial effects of positive youth development attributes and better family functioning on lower levels of pornography consumption among early Chinese adolescents [22]. The correlation results, though the magnitudes were not high, reinforced the notion that higher levels of positive youth development qualities would predict lower levels of youth health risk behavior [36, 37]. It is important to note that the link between one of the attributes, clear and positive identity and pornography consumption was not significant. This finding was inconsistent with previous results based on the same sample at secondary 1 [22]. Perhaps, this may be related to the unique characteristics of secondary 2 students. This observation underscores the importance of looking at the phenomenon using longitudinal data.

The present study attempted to explore the linkage between family functioning and pornography consumption. Three features of family functioning, mutuality, communication and harmony were negatively related to pornography consumption. Prior studies showed that parental monitoring, parental control, and quality of parent-child relationship were associated with the decrease of externalizing behaviors, delayed sexual intercourse, and substance use [38–41]. Our findings extend the pornography literature by providing useful additional evidence on the predictors of pornography consumption among Chinese adolescents. In addition, the observation for secondary 1 students was replicated for secondary 2 students.

One of the unique characteristics of the present study is the examination of pornography exposure among Chinese adolescents across two waves of data. Our results support the notion that there is an increasing tendency of pornography consumption with age. As most studies on pornography consumption were crosssectional in nature [13, 29, 35], little is known the developmental changes of pornographic materials consumption over time. Research in this area is important because risk and protective factors for pornography consumption might vary from one culture to another [20]. The current study overcomes these limitations by using a longitudinal design based on Chinese adolescents. It sheds light on designing appropriate programs for Chinese adolescents.

 Because our findings were based on Hong Kong Chinese adolescents, caution should be taken when they are generalized to other Chinese cultures, such as mainland China and Taiwan. Despite the emphasis on anonymity and confidentiality, social desirability and other biases cannot be eliminated in the study. Furthermore, the reliance on self-report data is another limitation. This might explain the low prevalence of pornography consumption as found in the study. However, this approach is commonly used in this field of research and underreporting is expected. Of course, the present findings require replication using a three-wave design to understand the related changes over time.

In the present study, the effect of early exposure to pornography was not examined. It would be desirable to investigate the potential harm of this premature exposure to pornography. As noted by Zillmann [12], “next to nothing is known about the consequences of the steadily increasing amount of such exposure” (p. 41). Clearly, more research in understanding the impact of this exposure among young people is warranted. Also, future study should include other factors, such as reasons for exposure, social context of use, and sexual socialization, which have been widely examined in previous pornography studies [42, 43].

Despite the above limitations, the present study basically replicated the correlates of pornography consumption among Hong Kong early adolescents. The proliferation of pornographic materials and the emergence of a “pornified” world [44] through the internet becomes an integral part of lives and contemporary cultures among adolescents [11]. Therefore, more efforts in promoting media literacy among this population should be emphasized. As positive youth development programs such as the Project P.A.T.H.S. can promote adolescent development and reduce adolescent risk behavior [45–49], it would be exciting to see whether such programs can reduce adolescent pornographic behavior. Furthermore, as pointed out by Hald [13], “future research (should) focus on these situational, interpersonal, interpersonal, and behavioral characteristics of pornography consumption in addition to actual prevalence rates of consumption in different cultures” (p. 584). With reference to this suggestion, the present study represents a positive response to this request and adds to the growing literature on pornography consumption. Furthermore, as mentioned by Hubbard and Vetter [50], “replication and extension research can play a major role in ensuring the integrity of a discipline's empirical results” (p. 153). The present study basically replicated the findings based on secondary 1 students.

## Figures and Tables

**Table 1:** Demographic information of the respondents (*N* = 3,638).

	*n*	%
Gender*		
Male	1,864	52
Female	1,716	48
Place of birth		
Hong Kong	2,806	79
Mainland China	690	19
Others	73	2
Location of schools		
Hong Kong island	5	18
Kowloon	7	25
New territories	16	57
Parents' marital status		
Married	2,985	83
Divorced/separated	334	9
Remarried	168	5
Others	122	3
Receiving financial aids		
Yes	208	6
No	2,932	81
Others	472	13

*58 respondents did not identify their gender.

**Table 2:** Past year exposure to internet and traditional pornographic materials.

	Never (%)	Less than 1 time a month (%)	1–3 times a month (%)	About 1 time a week (%)	Several times a week (%)	Daily (%)
Internet						
Pornographic stories	89.8	6.3	1.7	.8	.9	.6
Pornographic pictures (exposed genitals)	88.5	7.5	1.5	.8	1.0	.6
Pornographic videos (exposed genitals)	88.4	6.4	2.2	1.1	1.1	.7
Sexual intercourse pictures (including comics)	90.2	5.5	1.6	.9	1.2	.6
Sexual intercourse videos (including cartoons)	89.6	5.8	2.0	.9	1.0	.6
Pornographic website	91.5	4.9	1.3	.8	.8	.7

Traditional						
Pornographic movies	98.3	1.0	.2	.1	.1	.3
Pornographic rental films	98.5	.7	.2	.1	.1	.4
Pornographic movies on cable TV	97.7	1.3	.2	.1	.2	.5
Pornographic magazines	97.6	1.3	.3	.2	.2	.5
Pornographic books	96.7	1.8	.5	.2	.3	.5
Pornographic comics	95.0	3.0	.8	.2	.4	.5

**Table 3:** Past year exposure to internet and traditional pornographic materials by gender.

	Attempted (%)	
	Male	Female
Internet		
Pornographic stories	12.2	8.1
Pornographic pictures (exposed genitals)	16.6	6.0
Pornographic videos (exposed genitals)	17.6	5.4
Sexual intercourse pictures (including comics)	13.2	6.2
Sexual intercourse videos (including cartoons)	14.6	6.0
Pornographic website	3.1	3.1

Traditional		
Pornographic movies	2.4	1.0
Pornographic rental films	2.0	1.0
Pornographic movies on cable TV	2.9	1.6
Pornographic magazines	3.4	1.4
Pornographic books	4.0	2.7
Pornographic comics	5.2	4.7

**Table 4:** Correlations among variables in the model.

	*M* (SD)	*α* (mean)^#^	Overall pornography (*α* = .95)	Internet pornography (*α* = .96)	Traditional pornography (*α* = .94)
Subscales based on primary-order factors					
BO	4.60 (.90)	.77 (.53)	−.14**	−.12**	−.12**
RE	4.57 (.90)	.81 (.59)	−.11**	−.09**	−.10**
SC	4.67 (.89)	.88 (.71)	−.09**	−.08**	−.06**
PB	4.20 (.95)	.77 (.53)	−.14**	−.13**	−.11**
EC	4.27 (.90)	.74 (.49)	−.10**	−.10**	−.07**
CC	4.35 (.86)	.83 (.62)	−.11**	−.09**	−.10**
BC	4.49 (.82)	.76 (.52)	−.11**	−.10**	−.10**
MC	4.38 (.87)	.75 (.50)	−.14**	−.13**	−.10**
SD	4.41 (.88)	.78 (.55)	−.09**	−.08**	−.06**
SE	4.33 (.93)	.68 (.51)	−.06**	−.06**	−.04**
SI	4.06 (1.01)	.80 (.57)	−.02	−.02	−.02
BF	4.26 (1.06)	.85 (.66)	−.07**	−.07**	−.06**
PI	4.26 (.98)	.82 (.59)	−.11**	−.11**	−.07**
PN	4.50 (.95)	.72 (.47)	−.18**	−.18**	−.12**
SP	4.99 (1.30)	.89 (.73)	−.09**	−.09**	−.07**

Subscales based on second-order factors					
CBC	4.42 (.74)	.84 (.65)	−.12**	−.10**	−.10**
PA	4.38 (.86)	.75 (.60)	−.16**	−.16**	−.12**
GPYDQ	4.51 (.71)	.88 (.49)	−.15**	−.14**	−.11**
PID	4.16 (.96)	.84 (.73)	−.05**	−.05**	−.04*

Subscales based on family functioning					
Mutuality	3.80 (.90)	.88 (.72)	−.12**	−.12**	−.09**
Harmony	2.27 (.93)	.78 (.55)	−.10**	−.11**	−.09**
Communication	3.41 (.96)	.81 (.59)	−.09**	−.10**	−.06**

**P < .01; *P < .05.

Note. BO: bonding; RE: resilience; SC: social competence; PB: recognition for positive behavior; EC: emotional competence; CC: cognitive competence; BC: behavioral competence; MC: moral competence; SD: self-determination; SE: self-efficacy; CPI: clear and positive identity; BF: beliefs in the future; PI: prosocial involvement; PN: prosocial norms; SP: spirituality; CBC: cognitive-behavioral competencies second-order factor; PA: prosocial attributes second-order factor; GPYDQ: general positive youth development qualities second-order factor; PID: positive identity second-order factor.

**P < .01.

^#^Mean interitem correlations.

**Table 5:** The Chi-square test of independence among different types of pornographic materials by gender, immigrant status, and family economic background.

	Overall pornography		Internet pornography		Traditional pornography	
	%^*∧*^	*χ* ^2^	%^*∧*^	*χ* ^2^	%^*∧*^	*χ* ^2^
Gender						
Male	26	85.68**	24.4	85.64**	7.9	3.35**
Female	13		12.3		6.3	
Immigrant status						
Hong Kong	18.4	14.92**	17.2	12.24**	6.8	2.08
Mainland China	24.5		22.7		8.2	
Receiving financial aids						
No	23.1	1.63	21.5	1.46	7.7	.96
Yes	19.5		18.2		6.9	

**P < .01.

^*∧*^The observed percentage.

**Table 6:** Paired *t*-test results among different types of pornographic materials by wave.

	Wave 1	Wave 2	*t*	*d*
	*M* (SD)	*M* (SD)		
Overall pornography	1.05 (.22)	1.10 (.40)	−7.80**	.15
Internet pornography	1.08 (.32)	1.16 (.57)	−8.59**	.17
Traditional pornography	1.02 (.15)	1.04 (.29)	−3.96**	.09

**P < .01.
